# Automatic, context-specific generation of Gene Ontology slims

**DOI:** 10.1186/1471-2105-11-498

**Published:** 2010-10-07

**Authors:** Melissa J Davis, Muhammad Shoaib B Sehgal, Mark A Ragan

**Affiliations:** 1 The University of Queensland, Institute for Molecular Bioscience and ARC Centre of Excellence in Bioinformatics, Brisbane, QLD 4072, Australia; 2 Queensland Facility for Advanced Bioinformatics, The University of Queensland, Institute for Molecular Bioscience, Brisbane, QLD 4072, Australia; 3 Microsoft Corporation, One Microsoft Way, Redmond, WA 98052-8300, USA

## Abstract

**Background:**

The use of ontologies to control vocabulary and structure annotation has added value to genome-scale data, and contributed to the capture and re-use of knowledge across research domains. Gene Ontology (GO) is widely used to capture detailed expert knowledge in genomic-scale datasets and as a consequence has grown to contain many terms, making it unwieldy for many applications. To increase its ease of manipulation and efficiency of use, subsets called *GO slims *are often created by collapsing terms upward into more general, high-level terms relevant to a particular context. Creation of a GO slim currently requires manipulation and editing of GO by an expert (or community) familiar with both the ontology and the biological context. Decisions about which terms to include are necessarily subjective, and the creation process itself and subsequent curation are time-consuming and largely manual.

**Results:**

Here we present an objective framework for generating customised ontology slims for specific annotated datasets, exploiting information latent in the structure of the ontology graph and in the annotation data. This framework combines ontology engineering approaches, and a data-driven algorithm that draws on graph and information theory. We illustrate this method by application to GO, generating GO slims at different information thresholds, characterising their depth of semantics and demonstrating the resulting gains in statistical power.

**Conclusions:**

Our GO slim creation pipeline is available for use in conjunction with any GO-annotated dataset, and creates dataset-specific, objectively defined slims. This method is fast and scalable for application to other biomedical ontologies.

## Background

### Gene Ontology in annotation and analysis

The abundance of genome-scale data across the many species and biological contexts of interest to modern molecular biology and genetics has created substantial problems for data interoperation and integrated analysis, particularly when the creation and analysis of these data is highly distributed. Likewise, the rate of discovery in molecular genetics has accelerated rapidly, due in no small measure to new high-throughput genome-scale technologies and automation. Capturing new knowledge as annotation, and making it easily accessible to other research groups and to computational methods, will remain challenging so long as the primary storehouse of biological knowledge is natural-language statements collected in peer-reviewed scientific literature. An important way forward is through the adoption of controlled vocabularies that can be used to annotate collections of biological entities with statements that reflect the current state of biological knowledge about those entities. Controlled vocabularies, or ontologies, establish precise, agreed-upon definitions for terms, and establish the context in which those terms may be used. In this way ontologies facilitate the reuse and exchange of knowledge by researchers, and enable the broader application of computational methods over a vocabulary vastly reduced from that of natural language.

The most widely adopted of these controlled vocabularies is the Gene Ontology (GO; http://www.geneontology.org) [1]. GO covers knowledge about the molecular function of gene products, the biological processes in which they are active, and the cellular component in which they function or reside. By coupling GO terms with gene-product identifiers, the annotation process associates biological information with identifiers in formally defined machine-readable formats and thus enables the exchange, analysis and re-use of biological knowledge.

The Gene Ontology Consortium, which is responsible for the ongoing development of GO, draws its members from a number of organism-specific databases including FlyBase [2], Mouse Genome Database [3], WormBase [4], the Arabidopsis Information Resource [5], and the Zebrafish Information Network [6]. These consortium members, and others such as the Gene Ontology Annotation Database [7,8], produce GO annotations for public use. This community structure has contributed to the broad acceptance and adoption of GO as the primary controlled vocabulary for molecular genetics and genomics.

GO is commonly represented as a tree-like hierarchy of terms, in which each term can have child terms that are more-specific subclasses of the parent class. In fact, the GO hierarchy is a directed acyclic graph (DAG) rather than a tree, as a GO term can have multiple parents. Indeed, because GO covers three domains of knowledge and relationships are defined only within each knowledge type, GO is in fact three distinct, non-overlapping DAGs identified by their respective namespaces: biological_process (BP), molecular_function (MF), and cellular_component (CC).

Information about the cellular biological context of a gene product (*e.g*. protein or RNA) is extremely valuable, and GO annotations are used in a wide variety of domains and applications. For example, GO terms are commonly used to identify biological processes or molecular functions over- or under-expressed in different cell types, developmental states or disease conditions [9], identify likely false positives [10], evaluate predictive methods [11], and examine whether genes of like function are clustered along the genome [12]. The success of GO in these and many other contexts relies on the quality and currency of its annotation, and considerable resources must be invested by researchers and communities into its ongoing manual development and curation. Even with substantial coordination across these activities, equivalent parts of the GO graph end up being developed to very different degrees of resolution and detail.

The wide adoption of GO has contributed to the proliferation of terms within it, and as of April 2010 GO contained 18903 terms for biological processes, 8713 for molecular functions and 2734 for cellular components, excluding obsoletes. Its size contributes to its broad applicability, but makes it difficult for users to select GO terms for annotation, or to compare and analyse data annotated with GO terms. The hierarchical structure of GO establishes transitive relationships between terms: for any gene product annotated with a term, annotation with all the parents of that term, back to the root, must also be biologically sound. This property has been exploited to create smaller, more-manageable subsets of GO, called *GO slims*, that focus on terms relevant to a specific problem or data set. These GO slims can then be used to generate higher-level annotation more robust to tests of statistical significance [13].

A number of projects have created GO slims, seven of which are currently maintained by the GO Consortium http://www.geneontology.org/GO.slims.shtml. Two of these are general GO slims (Generic GO slim [14] and Gene Ontology Annotation (GOA) GO slim [15]), while four are domain-specific (Plant GO slim [16] and GO slims for three yeasts including *Saccharomyces *[17]) and one is specific to the Protein Information Resource http://pir.georgetown.edu/. Other GO subsets that have been archived but are not maintained include organism-specific slims for the honey bee *Apis mellifera*, the fruit fly *Drosophila melanogaster*, the malarial parasite *Plasmodium falciparum *and rice *Oryza sativa*. Tools exist for mapping between GO-slim sets and full GO annotation, such as the Map2slim application http://search.cpan.org/~cmungall/go-perl/ for which several web-based implementations exist (*e.g*. http://amigo.geneontology.org/cgi-bin/amigo/slimmer and http://go.princeton.edu/cgi-bin/GOTermMapper/GOTermMapper).

Efforts have been made to automate or semi-automate the creation of GO subsets. The OBO Edit application provides a graphical browser that can be used to mark terms for inclusion in a GO slim and check that complete paths exist between the selected terms and the root term of each graph. A taxonomy-based method has been developed to create species-specific subsets of GO [18], although it is not extensible to slim creation in general. To the best of our knowledge, however, no automated tool support currently exists for the creation of GO slims. In this paper we introduce a general approach, based on ontology management principles, graph theory and information theory, for the automated generation of ontology slims based on information obtained from both annotations and the ontology structure, and we illustrate the application of this method to the generation of high-quality GO slims at a series of information content thresholds. This framework also includes annotation management and semantic synchronisation features that reduce information lost as data lose currency and terms become obsolete.

### Graph and information theory as applied to GO

While GO may be thought of as a hierarchically ordered controlled vocabulary, it is topologically a directed acyclic graph (DAG). GO terms form the nodes (vertices) in this graph, and relationships between terms form the edges. Directionality on the edges is established by the *is_a *and *part_of *relations that are transitive in nature, and establish more-specific terms as sub-classes of more-general terms, and progressively group information up the hierarchy to the root term of each graph: biological_process (GO:0008150), cellular_component (GO:0005575), and molecular_function (GO:0003674) respectively. Of the two relationship types, *is_a *relationships establish conceptual subclass-superclass relationships between terms, while *part_of *relationships establish a subset-superset relationship. This information is contained in the graph structure itself, while additional information is contained in the gene-product annotations.

The creation of GO slims, therefore, must carefully reduce the information loss, from the perspectives of both graph structure and gene-product annotation. Information content can be computed from the distribution of GO terms in annotated datasets, from the structured relationships between GO terms in the ontology itself, or from both sources in combination. For example, Wang *et al. *[19] used semantic similarities of GO terms to find functional similarities of genes by introducing weights for the different relations, while Tao *et al. *[20] introduced semantic similarity for gene-function prediction based on the node's location and semantic relationships [21]. Other approaches have also been based on topological measures of similarity, such as the shortest path between terms, to determine similarity between GO terms [22].

Resnik [23,24] developed an information theoretic measure of similarity based on the probabilities of co-occurring terms in a set of instance data (equivalent here to a dataset of gene products annotated with GO terms), in order to avoid the unreliability of topology alone in calculating term similarity. Similarly, using information available from annotated gene product datasets, Del Pozo and colleagues [25] introduced a GO similarity measure based on the simultaneous occurrence of GO terms in a curated dataset from InterPro [26].

Thus although both graph theory and information theory have been explored to gain biological insight from datasets annotated using GO, and a number of GO slims have been created and are being maintained for use by research communities, to the best of our knowledge no tool support currently exists for the automatic generation of GO slims. Here we introduce such a method that can generate customised GO slims for specific annotated datasets. Our method finds an optimal reduced GO graph by penalising graph complexity, while at the same time minimising information loss (coverage) by retaining terms with high information-content values. We compute the information content of a term based both on its position in the GO DAG and on the gene-product annotations associated with it in a given annotated dataset, while taking into account the information lost if the term is removed. In this our method differs from classical information-theoretic approaches that compute information content without reference to graph structure [23-25].

## Results

### GO slim for yeast

Here we analyse a set of GO slims generated across a range of information content thresholds on the yeast GO annotation contained in the SGD database [27], and compare them with the manually created yeast GO slim maintained by the yeast community. We examine the composition of slims created by our method over a range of information thresholds, and finally we examine the improvements in statistical power that result from conducting functional enrichment analysis based on GO slim terms compared with full GO annotation.

**Figure 1:**
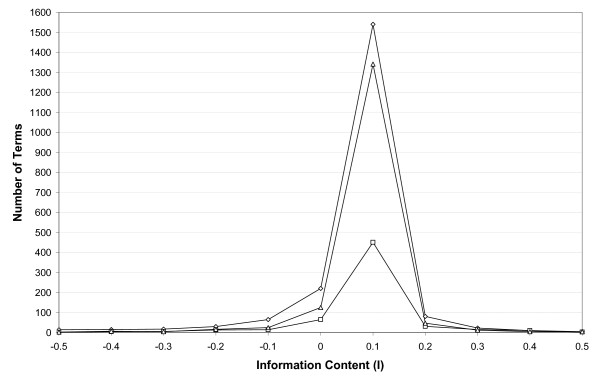
**Frequency distribution of Information Content (I) values obtained for BP (triangle), CC (square) and MF (diamond) terms annotated in the SGD Yeast data**. The number of terms with values of I between -0.5 and 0.5 is shown, and includes 96.6% of BP terms, 96.3% of CC terms, and 97.5% of MF terms.

As expected, when examining GO slims generated at a range of thresholds, we observe that progressively raising the information content threshold yields slim ontologies of reduced complexity, with fewer terms included in the slim subset (Table 1). Selection of an optimal threshold for creating a slim will depend on the intended use(s) of that slim, and on the level of resolution desired in the resulting GO slim file. The frequency of information content values *I *obtained for the input terms can provide some guide to selecting an appropriate value for the threshold, *τ*. For example, Figure 1 shows the frequency distribution of *I *for the GO CC terms in the yeast dataset. Most have values between 0.0 and 0.1, while Table 1 shows that the number of terms selected becomes relatively stable for values over 0.5 because few terms hold values of *I *over this threshold.

**Table 1:** Size of the slim ontology generated across a range of thresholds on the Yeast SGD GO annotation data.

IC Threshold (τ)	Biological Process	Cellular Component	Molecular Function	Total size of GO slim
0.0	2634	700	1886	5220
0.1	579	189	215	983
0.2	286	120	98	503
0.3	114	82	74	270
0.4	57	58	59	174
0.5	41	53	48	142
0.6	33	53	42	128
0.7	33	46	42	121
0.8	33	46	39	118
0.9	26	46	34	106
1.0	22	46	34	102

Above thresholds of 0.5, the size of the GO slim subset changes little.

**Figure 2:**
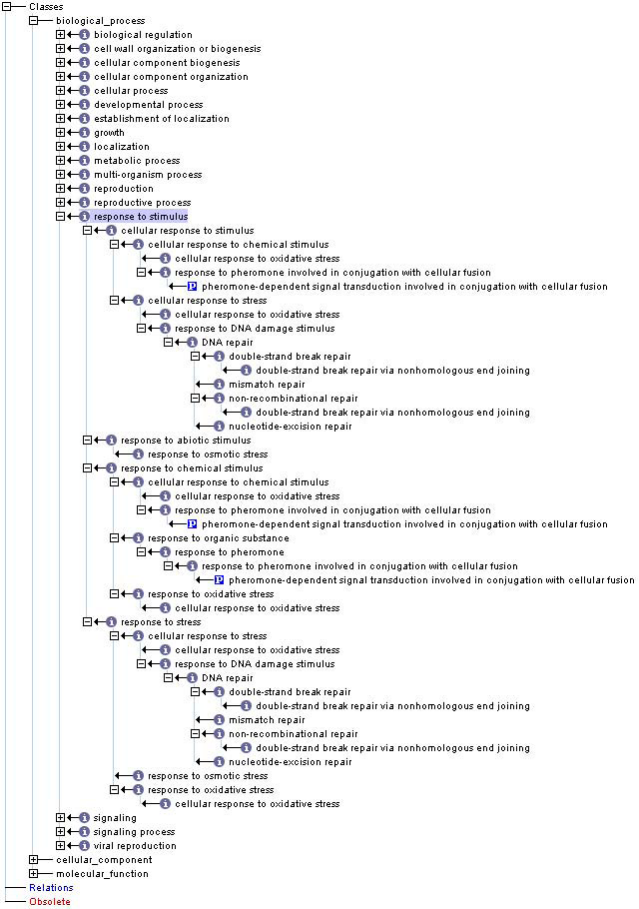
**GO slim generated using an information content threshold of 0.1, showing the top level of the Biological Process hierarchy with full expansion of the children of *response to stimulus***.

**Figure 3:**
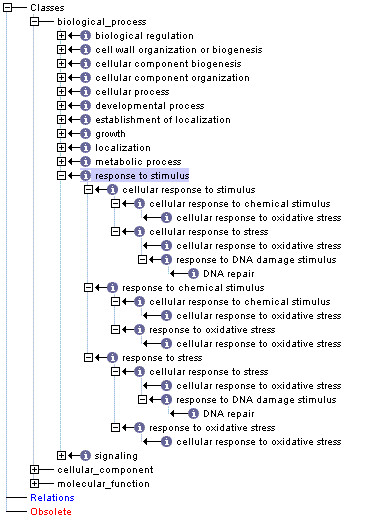
**GO slim generated using an information content threshold of 0.2, showing the top level of the Biological Process hierarchy with full expansion of the children of *response to stimulus***.

**Figure 4:**
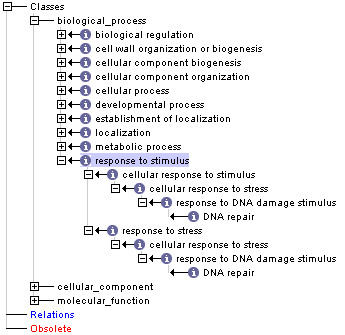
**GO slim generated using an information content threshold of 0.3, showing the top level of the Biological Process hierarchy with full expansion of the children of *response to stimulus***.

In additional files 1, 2 and 3, we provide full mappings between annotation terms and selected slim terms for three values of *τ*: 0.1, 0.2 and 0.3. To illustrate the performance of the method at these thresholds, here we examine in detail the mapping of terms within the top level of the BP namespace (the immediate children of *biological_process *(GO:0008150)), focusing on the descendents of *response to stimulus *(GO:0050896) at each threshold (Figures 2, 3 and 4). This example highlights the trade-off between reducing the complexity of the GO slim graph, and maintaining detail in the associated annotation.

It is noteworthy that using our approach and scripts, slims are automatically generated for the yeast dataset in less than a minute on a standard system (Red Hat Enterprise Linux 4 (32 bit), 2x 2.6MHz vCPUs, 1GB RAM). With this speed of execution, researchers can easily experiment with a range of *τ *values to achieve their desired level of resolution and conceptual depth.

*Response to stimulus *has 20 descendents in the GO slim generated using *τ *= 0.1 (Figure 2), nine descendents at *τ *= 0.2 (Figure 3), and five at *τ *= 0.3 (Figure 4). The descendent count for *response to stimulus *then remains stable between 0.3-0.5 as child term *DNA repair *has a information content of 0.52. Gene products annotated with specific terms not included in the slim are collapsed upward to the most-specific terms retained. Thus a term originally annotated with GO:0019236 (*response to pheromone*) would retain this annotation if mapped to a slim generated at *τ *= 0.1, would be collapsed to the term GO:0042221 (*response to chemical stimulus*) at *τ *= 0.2, and would be collapsed to GO:0050896 (*response to stimulus*) at *τ *= 0.3-0.5.

*Biological process*, which has 32 immediate children in the full ontology, has 17 immediate children at τ = 0.1 (Figure 2), 12 at τ = 0.2 (Figure 3), and 10 at τ = 0.3 (Figure 4) and five at τ = 0.5. Progressive loss of immediate children of *biological_process *results in an increasing number of terms being mapped back to the root node: while only 10 terms are collapsed non-specifically to the root node at the lower threshold, 105 terms are collapsed to the root node at τ = 0.2, and 125 at τ = 0.3 (for details see additional files 1, 2 and 3). This illustrates the loss of resolution inherent in creation of small, high-level GO subsets. While gene products whose GO annotations are collapsed to the root node lose their detailed annotation, annotation with the root node label at least retains the association of the gene product with a known (although unspecified) biological process. The extent of collapse can be prioritised on the basis of an information-content calculation and is therefore an objective, rather than subjective, process. Finally, the outcome is repeatable, not dependent on the vagaries of individual or collective human decisions.

### Comparison of results with manually produced GO slim

**Figure 5:**
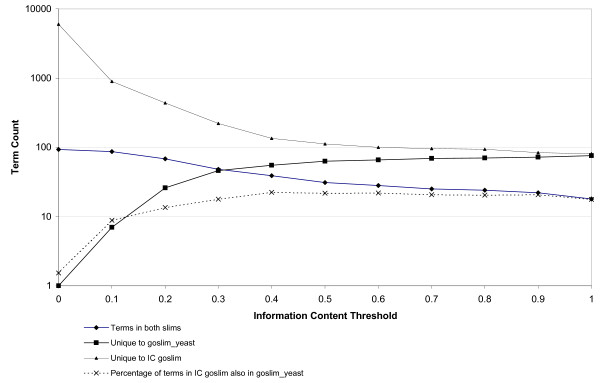
**Comparison of the goslim_yeast maintained by SGD with the GO slim generated by our method across a range of threshold values**. Threshold τ is given on the x axis, while the y axis presents the number of GO terms according to a log scale. The dotted line represents the percentage of terms in the IC goslim that are also found in the SGD goslim yeast set (minimum: 1.5%, maximum 22.4%).

We compared the GO slims generated by applying our automated method on the SGD protein annotations to the one produced manually by curators associated with the SGD database, to explore the extent to which our process retrieves terms considered important by human experts. The SGD yeast GO slim (goslim_yeast) is made available to the community through the Gene Ontology Consortium website as one of the set of GO slims maintained at http://cvsweb.geneontology.org/cgi-bin/cvsweb.cgi/go/GO_slims/ and includes 94 terms. Our slims range in size from 6088 to 102 terms (Table 1). We present the results of this comparison over a range of information content thresholds in Figure 5.

The maximum overlap between the subsets is found at the lowest threshold value (τ = 0.0), where all but one goslim_yeast term is included. The missing term, *spindle envelope *(GO:0070732), is not included in our subset because it is not used to annotate any gene product in the set of yeast proteins from SGD. At progressively higher values of τ fewer overlapping terms are observed; the proportion of overlapping terms, computed as a proportion of total terms in our GO slim, peaks at ~22% at τ = 0.4.

While our automatically generated GO slims never achieve full overlap with the manually created subset, we contend that this is due to bias in the selection of terms by human curators. For example, no term exists in the SGD yeast GO slim corresponding to the CC concept *integral to membrane *(GO:0016021) despite the fact that around one quarter of all yeast proteins are likely to be integral to a membrane [28], and > 1500 SGD proteins are annotated with this term. *Integral to membrane* has functional implications distinct from those of the parent term *membrane *(GO:0016020), which is included in both slims. Likewise, the molecular function term *ATP binding *(GO:0005524) is used to annotate > 1100 yeast proteins, but is absent from the yeast slim. Terms such as *protein complex biogenesis *(GO:0070271), *cellular component morphogenesis *(GO:0032989) and *generation of precursor metabolites and energy *(GO:0006091) that are present in the yeast_slim, but are either not used to annotate gene products, or used only once, will not appear in our objectively defined slims, or will appear (in the case of terms used only once) only at the most permissive of thresholds.

### Case study: functional enrichment analysis

To evaluate how effectively our method improves the clarity of functional enrichment analysis, we use the hypergeometric test as previously applied [29,30] to identify GO terms significantly enriched in a set of differentially expressed genes identified through microarray profiling of yeast sporulation [31]. The SGD Expression Connection interface was used to retrieve 341 genes with greater than a five-fold increase in expression during yeast sporulation. The genes were mapped to SGD accession numbers, and 339 unambiguously mapped genes and associated gene ontology annotations are available in additional file 4.

Because many GO terms occur in the gene lists and each term is tested individually, multiple hypothesis testing (MHT) correction methods such as the Bonferroni method [32] or Benjamini-Hochberg false discovery rate (FDR) correction [33] should be applied. Here we compare the results of the hypergeometric test before and after mapping genes in the list to a GO slim generated at τ = 0.3 for that experiment. In this sporulation experiment, 3654 genes have GO annotations of biological process, as do 206 of the 339 genes identified in our Expression Connection search. While 1578 GO BP terms were used to annotate the full set of yeast gene products, after mapping to the τ = 0.3 GO slim, only 177 GO BP terms are required.

The p-values for equivalently ranked GO terms are presented in Table 2, and demonstrate the benefit of reducing the number of hypotheses (*i.e*. GO terms) tested.

**Table 2:** Selected p-values with corresponding multiple hypothesis corrections, with significant values in bold.

Rank order according to p-value	Hypergeometric test p-values		Bonferroni-corrected p-values		Benjamini-Hochberg-corrected p-values	
	
	Full GO	GO slim	Full GO	GO slim	Full GO	GO slim
1	**0.0000**	**0.0000**	**0.0000**	**0.0000**	**0.0000**	**0.0000**
20	**0.0001**	**0.0002**	0.1578	**0.0354**	**0.0079**	**0.0018**
40	**0.0065**	**0.0104**	10.257	1.841	0.2564	**0.0462**

These results demonstrate that application of our method at τ = 0.3 decreases the Bonferroni correction by nearly ten-fold: using the full GO annotation set, p-values are multiplied by 1578, while using our GO slim, p-values are multiplied by only 177. With the more-permissive Benjamini-Hochberg correction, only the first 27 terms (those with the best p-values) from the full GO annotation tests retain significance after correction, compared with 40 terms from the GO slim. Balanced against this increased statistical power is the reduced detail available from the terms that remain. In this example, terms covering *mitotic spindle elongation *and *mitotic sister chromatid segregation *were found to be significant in the full annotation set, but were collapsed to *mitotic cell cycle *in the GO slim.

### Availability

This method has been implemented as a computational pipeline and is available for download from the Tools and Data page at http://bioinformatics.org.au. Example data and a user's guide are included with this download.

## Discussion

We have introduced a method, based on graph and information theory, for the automatic generation of ontology subsets (slims). Given an ontology and a particular set of annotations generated using that ontology, ontology slims optimally matched to those terms can be generated at any permissible threshold of information content. Our method has potential to supplement, or indeed replace, the laborious, error-prone and sometimes idiosyncratic manual creation and curation of ontology slims for specific species, problems or research areas.

Our method maximises the information content available in the resulting slim, while minimising its size. Our node information content metric, *I_n_*, incorporates information from both the graph structure and the annotated data. Existing methods are unsuited for calculating information content relevant to ontology-subset selection; Shannon's mutual information, for example, considers conditional dependency of nodes but not graph complexity. It was our intention to construct slims that concomitantly minimise both information loss (through loss of detail in annotation when specific terms are removed) and the number of terms used. Likewise, methods that calculate the information of terms based only on graph structure neglect the context-specific information contributed by specific sets of annotated gene products, and as such are unsuitable for creating ontology subsets specific to a given biological dataset.

Our approach begins with an annotation processing phase in which we update the annotation file by identifying and resolving inconsistencies between the annotation and the current version of the GO. Inconsistent terms that can be clearly mapped to a new term in the ontology are updated, while annotations using terms entirely removed from the ontology, or without clear successors, are removed to a separate file for manual resolution. This process ensures that information contributed by old annotations is preserved and updated, and removed only if corresponding concepts no longer have equivalents in the current GO. These pre-processing methods are of broader applicability, for example in updating historical annotation datasets, or automatically preserving currency in existing annotated datasets.

Our method for creating GO slims is automatic and fast, generating slims that are complete (each slim covers all the terms used in annotating the given set of gene products), well-formed (the subgraphs abide by the rules for constructing GO, and all terms have at least one complete path to the root, *i.e*. orphan terms are absent), and objectively constituted (each slim contains only the terms with information content greater than a user-defined threshold, and those additional terms required for completeness of paths). These desirable properties are not necessarily shared by manually curated slims, as illustrated by our analysis of goslim_yeast. Our information-content metric takes into account both graph structure and annotation, and as a consequence annotation is preserved at hierarchical levels such that information content is maximised across the entire annotation set. All gene products are guaranteed to map to a term in the slim, although in the worst (least-informative) case this may be the root term of a namespace. These properties enable users to balance generality against specificity, tuning the slim to a granularity appropriate for each individual problem or application.

Using this approach, small groups or individuals can create and maintain high-quality customised ontology slims, and keep these slims up to date with respect to emerging gene-product datasets and the most recent version of GO. Presently, the lack of tool support available for GO slim construction means that the process of creating and maintaining a GO slim is arduous, and while many slims are created, fewer are maintained (see GO website of archived slims). These custom slims preserve relationship types (*is_a*, *part_of*), and can be flexibly tailored to storage or computational resources. Like other slims, our custom slims offer greater statistical power than full ontologies, as a reduction in the number of hypotheses lessens the impact of MHT correction.

Although here we illustrate our method only in application to GO, it is directly applicable to any ontology presented in the Open Biomedical Ontology (OBO) format. Many ontologies are available in this format, covering such areas as organismal anatomy, taxonomy, mass spectrometry and chemical entities. A list of OBO ontologies is available at http://www.obofoundry.org/. Our method is more-generally applicable to any ontology with a DAG structure, and for which the relations between terms are transitive and a corresponding annotated dataset exists. The speed of the method over GO also indicates that our approach will support the generation of slims from ontologies much larger than GO.

## Conclusion

Our ontology-engineering method enables researchers to create and maintain an automatically generated GO slim for a specific dataset of interest. To select informative terms objectively, we have developed a new information contents metric that combines information contained in the GO structure with that obtained from annotated datasets. By removing the time consuming and subjective ontology editing procedure previously required for the creation of a GO slim (and associated maintenance overheads of maintaining the currency of the slim in the face of continuing growth and updating of GO), a significant barrier to the use of engineered GO subsets is removed.

## Methods

### Data sets

The Saccharomyces Genome Database (SGD: http://www.yeastgenome.org/) maintains a set of proteins annotated with GO terms in the GOA format. We downloaded these annotations to assess the ability of our pipeline to manage obsolete annotations. The Gene Ontology website also maintains an updated version of the yeast annotation data. We downloaded the gene association file (Revision 1.1508, GOC Validation date: 14^th ^August, 2010) from that site to constitute an application dataset for this research. An earlier file, (Revision 1.1398) was used to test the management of obsolete terms in annotation (see Section 4.3.1, data not shown). SGD also maintains the Yeast GO slim (provided by the Gene Ontology Consortium at http://www.geneontology.org/GO.slims.shtml), which we downloaded for comparison against subsets created automatically by the process described below.

Yeast gene-expression data and analyses [31] were extracted from the SGD Expression Connection interface http://www.yeastgenome.org/cgi-bin/expression/expressionConnection.pl using the option *Search III: Find all genes that change expression in a manner you define in one or more datasets*. We retrieved *S. cerevisiae *genes that showed a five-fold increase in expression, or greater, in sporulation. A GO slim for this experiment was generated using the method and tools we describe here. This analysis was performed on the GO (Revision 1.1398), which is archived with our method at http://bioinformatics.org.au.

### Tools

OBO Edit was used to view the Open Biomedical Ontology (OBO)-formatted ontology files for GO, and to display the terms selected for the GO slim subset. OBO Edit can be downloaded from http://oboedit.org/ and documentation is available on that website.

MATLAB was used to perform the hypergeometric tests on these data, and to apply the Bonferroni and Benjamini-Hochberg false discovery rate (FDR) corrections for multiple hypothesis testing.

### Ontology Engineering Methods

#### Annotation management

The time and effort required to annotate datasets renders them valuable resources, and their re-use within or across research communities could be highly desirable. Once a dataset has been annotated with GO associations, however, it rapidly becomes out-of-date as GO is itself further revised and updated. As a result, terms used in annotating a dataset may become obsolete, and attempts to map such terms to up-to-date GO slims frequently result in loss of information associated with such an annotation. To address the issue of obsolete annotation terms in datasets, we developed pre-processing steps that detect inconsistencies between ontology versions, and create updated annotation files. We first identify terms used in the data annotation but not used as primary identifiers in the most-recent version of GO, and extract entries for these terms from the GO OBO file (where entries for all terms used historically in the ontology are maintained). We consider five classes of inconsistency between ontology versions: 1) alternative identifier usage for a current term; 2) obsolete term replaced with a current term; 3) obsolete term with a suggested replacement term; 4) obsolete term with multiple suggested replacements; and 5) obsolete term with complete removal of the concept.

Inconsistencies of the first two types can be resolved automatically by replacing an alternative identifier with the primary identifier, or by replacing the obsolete term identifier with the replacement term identifier. Inconsistencies of types 3 and 4 are removed from the annotation file and flagged for supervised resolution, while type 5 inconsistency is resolved by removing the obsolete annotation. A new annotation file is then written. This file can be used to generate a GO slim set using terms mapped to the current version of GO, or used in its own right as an updated annotation file.

#### Term selection and sub-graph definition

Terms used in the annotation of gene products are selected separately for each of the three component graphs of GO. The paths from these terms back to the root of each respective component graph are then calculated from the ontology definition provided in the GO OBO definition file, using only the transitive relations *is_a *and *part_of *as defined in that file. Relations of other types (such as *regulates*) are not used because they do not establish transitive relationships between terms. These paths define the sub-graph of each GO graph, *G_bp_*, *G_cc_*, and *G_mf_*, implicit in the annotation of the dataset under analysis.

Once the sub-graphs supporting the annotation dataset have been extracted, information on gene-product annotation present in the annotation file is transferred to the sub-graph: each term in the sub-graph is associated with a count of the number of gene products annotated with that term. In this way, the sub-graph is annotated with the information contained in the gene-product annotation file, unifying information from the graph structure with that from the annotations. These annotated subgraphs are used to calculate the information contents *I *of each term (Section 0).

#### GO slim creation and visualisation

**Figure 6:**
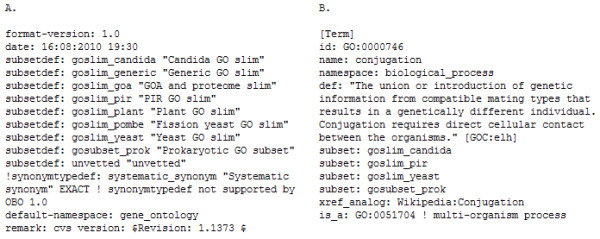
**Specification of GO slim subsets in the GO OBO file: (A) GO slim subsets are initially specified with a subsetdef: statement in the header; (B) each individual term that is a member of the GO slim is annotated with a subset: property in the term definition statement**.

Once the set of GO terms with information content *I *> τ is selected (Section 0), the set of all paths leading to these terms in each full GO graph is selected. The terms with *I *> τ and associated path terms are then annotated in the Gene Ontology OBO file as a defined subset. This subset is established with a subset definition line in the file header (Figure 6A) and each term that is a member of the GO slim is annotated with a subset membership property (Figure 6B).

**Figure 7:**
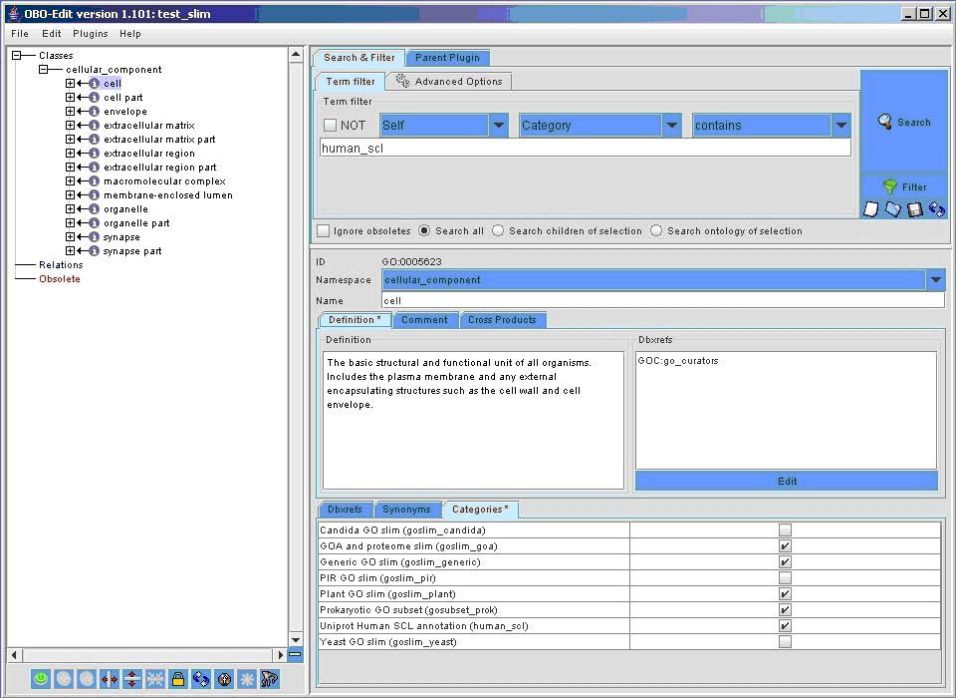
**Selection of a specific GO slim subset in the OBO Edit application: screen shot from OBO Edit showing the term filter set to view the human_scl category**. The Search & Filter tab is selected in the top right panel, and the term filter is set to select terms for which the category contains human_scl. The term *cell *is highlighted in the viewing panel on the left, and the term definition and other information (e.g. category membership) is displayed on the right. Categories to which a term belongs are ticked.

Once terms are annotated as members of a subset, the subgraph defined by membership of this set may be viewed in OBO Edit by using a term filter to select all terms that are members of the specified subset (Figure 7). OBO Edit can also be used to create, define and specify categories manually through the GUI, or to manually curate the sets of terms included in any specific GO slim http://oboedit.org/docs/index.html.

#### Mapping annotation to GO slim

**Figure 8:**
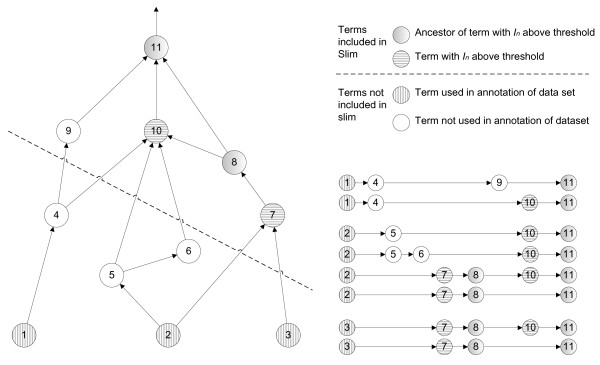
**DAG fragment to illustrate mapping between terms used in annotation to terms selected for GO slim using all paths through the graph to the root term**. In this DAG fragment, terms 1-3 are used to annotate gene products, and term 11 represents the root of the graph fragment. Paths from terms used in annotating gene products back to the root term are used to map from the full graph to the slim graph. All possible mappings are created, and account for the kinds of relations (either *is_a*, or *part_of*) used to construct the graph.

Once a GO slim is created, the original set of annotated gene products must be mapped to the high-level terms in the slim. This mapping is generated by collapsing the terms originally used in annotating the data set upward to the most-specific terms available in the slim. For example, consider the DAG fragment presented in Figure 8: terms 1-3 have been used to annotate a set of gene products and all terms 1-10 have node information values calculated according to equations 1-4; terms 7 and 10 have information content values exceeding the selected threshold, and therefore terms 7 and 10, along with terms 8 and 11, are selected for inclusion in the GO slim subset derived from the annotated data.

Annotation terms are mapped to the slim term(s) in closest proximity to the annotation term in the path(s) from the annotation term to the root node. As shown in Figure 8, term 1 would be mapped to terms 10 and 11, term 2 would be mapped to terms 7 and 10, and term 3 would be mapped to term 7. The mapping algorithm also distinguishes the type of relationship that exists between the annotation term and the slim term to which it is mapped: if they are connected by paths that contain only *is_a *relations, then the relationship between the annotation term and a mapped slim term is assigned as *is_a*. On the other hand, if the path contains at least one *part_of *relation, then that mapped relationship is assigned as *part_of*.

### Calculating information content of a term

The information content *I *of each term *n *in each sub-graph *G *(as defined in the process described above in *Term selection and sub-graph definition*) is computed using

(1) $$I_{n}=P_{n}-\Theta_{n}$$

where *P*_*n *_represents information gained if node *n *is retained in the graph, and Θ*_n _*is a penalty term that penalises node *n *for the information lost (by loss of coverage) if the children of term *n *are collapsed to *n *(*i.e*. child terms removed from the ontology). The term *information content *used here is different from that commonly encountered in information theory; in this context, information content of a node refers to information carried by a node based on its annotation and its position within the DAG, as described above.

*P_n _*of *n *is calculated as

(2) $$P_{n}=\lambda \frac{\beta_{n}}{\sum_{i=1}^{t}A_{i}}$$

where *t *is the total number of terms in the subgraph *G*, and *A_i _*is the annotation count attached to each node *i*. Due to sparseness of the ontology graph, the information content values can be very small. For convenience, we introduce a constant *λ *by which the information-content values are rescaled; here we use λ = 100.

*β_n _*is calculated such that

(3) $$\begin{array}{l} \beta_{n}=0\;\forall \;n\in L\\ \beta_{n}=A_{n}+\sum_{i=1}^{\zeta }\beta_{i}\;\forall \;n\notin L\;and\;n\in G \end{array}$$

where *A_n _*is the annotation count attached to *n*, *ζ *are the immediate children of node *n, β_i _*is the annotation count associated with child *i *of *n*, and *L  *϶ *L*⊂*G *is a set of leaf nodes which have no children. As indicated in the first part of equation 3, in the case where the node is a leaf node, *β_n _*is equal to zero.

The penalty term (information loss) Θ*_n _*is computed as

(4) $$\begin{array}{l} \Theta_{n}=0\;\forall \;\alpha =\text{0}\\ \Theta_{n}=\lambda \sum_{i=1}^{\alpha }P_{i}\log_{k}(\alpha )\;\forall \;\alpha \neq \text{0} \end{array}$$

where *P_i _*is computed for each child *i *of *n *using (2), and *α *is the number of children of node *n*. After computing *I *for each node, a threshold *τ *is applied to *I *to select a set of nodes. Note that we use log base *k*, where *k *is the number of children of the root node. The *λ *in (4) is the same constant used in (2) following the same rationale.

Values of *I_n _*as defined here can range between [-1,1] when *λ *= 1. A node will have maximum information content value if it is the only node in the graph: in that case *P_n _*= 1 and Θ*_n _*= 0, hence *I_n _*= 1. On the other hand, information content has minimum value in a 2-node graph where the parent node has no annotation attached to it while the child node has annotations; then the *P_n _*for the parent node will be zero while Θ*_n _*= 1 because *P_i _*= 1 and log_1_(1) = 1 in (4). We selected log base *k *in (4) to provide a minimum limit to the information content values, as just shown. Also, using log base *k *provides flexibility for information-content values to be adapted to the size of the graph.

A detailed description of the algorithm implementing these calculations over the GO sub-graphs is presented in additional file 5 (Supplementary Methods). A set of calculations on an example ontology graph is also provided in this file.

## Abbreviations

(BP): Biological Process namespace; (CC): Cellular Component namespace; (DAG): Directed Acyclic Graph: Gene Ontology; (MF): Molecular Function namespace; (OBO): Open Biomedical Ontology.

## Authors' contributions

MJD, MSS and MAR originated the project. MJD and MSS designed and evaluated the framework reported here. MSS developed and implemented the information content criterion and algorithms. MJD developed and implemented the ontology engineering workflow, wrote the user documentation, prepared code for release, organised datasets, performed the experiments, and analysed the results. All authors wrote the manuscript.

## Supplementary Material

Additional file 1 **GO slim at *τ *= 0.1 mapped to yeast annotation terms**. This file contains GO slim terms mapped to the full set of GO terms used in the GOA annotation of yeast. The GO slim was generated using a threshold *τ *= 0.1 Click here for file

Additional file 2 **GO slim at *τ *= 0.2 mapped to yeast annotation terms**. This file contains GO slim terms mapped to the full set of GO terms used in the GOA annotation of yeast. The GO slim was generated using a threshold *τ *= 0.2. Click here for file

Additional file 3 **GO slim at *τ *= 0.3 mapped to yeast annotation terms**. This file contains GO slim terms mapped to the full set of GO terms used in the GOA annotation of yeast. The GO slim was generated using a threshold *τ *= 0.3. Click here for file

Additional file 4 **SGD Expression Connection results for genes with increased expression in yeast sporulation**. This file contains genes retrieved from the SGD Expression Connection interface with greater than a five-fold increase in expression during yeast sporulation. Associated gene ontology annotations are also contained in this file. Click here for file

Additional file 5 **Supplementary Methods**. This file contains implementation details for the information contents calculation algorithm. Click here for file

## References

[B1] GeneOntologyConsortium The Gene Ontology (GO) database and informatics resource Nucleic Acids Research 2003 32 Database D258 D261 10.1093/nar/gkh036 PMC308770 14681407

[B2] Wilson RJ Goodman JL Strelets VB Gelbart W Bitsoi L Crosby M Dirkmaat A Emmert D Gramates L Falls K FlyBase: Integration and improvements to query tools Nucleic Acids Research 2008 36 Database D588 D593 18160408 10.1093/nar/gkm930 PMC2238994

[B3] Bult C Eppig J Kadin J Richardson J Blake J Airey M Anagnostopoulos A Babiuk R Baldarelli R Baya M The Mouse Genome Database (MGD): Mouse biology and model systems Nucleic Acids Research 2008 36 Database D724 D728 18158299 10.1093/nar/gkm961 PMC2238849

[B4] Rogers A Antoshechkin I Bieri T Blasiar D Bastiani C Canaran P Chan J Chen WJ Davis P Fernandes J WormBase 2007 Nucleic Acids Research 2008 36 Supplement 1 D612 617 17991679 10.1093/nar/gkm975 PMC2238927

[B5] Huala E Dickerman AW Garcia-Hernandez M Weems D Reiser L LaFond F Hanley D Kiphart D Zhuang M Huang W The Arabidopsis Information Resource (TAIR): a comprehensive database and web-based information retrieval, analysis, and visualization system for a model plant Nucleic Acids Research 2001 29 1 102 105 10.1093/nar/29.1.102 11125061 PMC29827

[B6] Sprague J Bayraktaroglu L Clements D Conlin T Fashena D Frazer K Haendel M Howe DG Mani P Ramachandran S The Zebrafish Information Network: the zebrafish model organism database Nucleic Acids Research 2006 34 Suplement 1 D581 585 10.1093/nar/gkj086 16381936 PMC1347449

[B7] Camon E Magrane M Barrell D Binns D Fleischmann W Kersey P Mulder N Oinn T Maslen J Cox A The Gene Ontology Annotation (GOA) Project: Implementation of GO in SWISS-PROT, TrEMBL and InterPro Genome Research 2003 13 4 662 672 10.1101/gr.461403 12654719 PMC430163

[B8] Camon E Magrane M Barrell D Lee V Dimmer E Maslen J Binns D Harte N Lopez R Apweiler R The Gene Ontology Annotation (GOA) Database: Sharing knowledge in Uniprot with Gene Ontology Nucleic Acids Research 2004 32 Database D262 D266 10.1093/nar/gkh021 14681408 PMC308756

[B9] Cortón M Botella-Carretero JI Benguria A Villuendas G Zaballos A San Millán JL Escobar-Morreale HF Peral B Differential gene expression profile in omental adipose tissue in women with polycystic ovary syndrome The Journal of Clinical Endocrinology and Metabolism 2007 92 1 328 337 10.1210/jc.2006-1665 17062763

[B10] Mahdavi M Lin Y-H False positive reduction in protein-protein interaction predictions using gene ontology annotations BMC Bioinformatics 2007 8 1 262 10.1186/1471-2105-8-262 17645798 PMC1941744

[B11] Jensen LJ Gupta R Staerfeldt HH Brunak S Prediction of human protein function according to Gene Ontology categories Bioinformatics 2003 19 5 635 642 10.1093/bioinformatics/btg036 12651722

[B12] Yi G Sze S-H Thon MR Identifying clusters of functionally related genes in genomes Bioinformatics 2007 23 9 1053 1060 10.1093/bioinformatics/btl673 17237058

[B13] Rhee SY Wood V Dolinski K Draghici S Use and misuse of the gene ontology annotations Nature Reviews Genetics 2008 9 509 515 10.1038/nrg2363 18475267

[B14] Harris M Clark J Ireland A Lomax J Ashburner M Foulger R Eilbeck K Lewis S Marshall B Mungall C The Gene Ontology (GO) database and informatics resource Nucleic Acids Research 2004 31 D258 261 10.1093/nar/gkh036 PMC308770 14681407

[B15] Biswas M O'Rourke JF Camon E Fraser G Kanapin A Karavidopoulou Y Kersey P Kriventseva E Mittard V Mulder N Applications of InterPro in protein annotation and genome analysis Briefings in Bioinformatics 2002 3 3 285 295 10.1093/bib/3.3.285 12230037

[B16] Berardini TZ Mundodi S Reiser L Huala E Garcia-Hernandez M Zhang P Mueller LA Yoon J Doyle A Lander G Functional annotation of the arabidopsis genome using controlled vocabularies Plant Physiology 2004 135 745 755 10.1104/pp.104.040071 15173566 PMC514112

[B17] Weng S Dong Q Balakrishnan R Christie K Costanzo M Dolinski K Dwight SS Engel S Fisk DG Hong E Saccharomyces Genome Database (SGD) provides biochemical and structural information for budding yeast proteins Nucleic Acids Research 2003 31 1 216 218 10.1093/nar/gkg054 12519985 PMC165501

[B18] Kuśnierczyk W Taxonomy-based partitioning of the Gene Ontology Journal of Biomedical Informatics 2008 41 282 292 10.1016/j.jbi.2007.07.007 17921072

[B19] Wang JZ Du Z Payattakool R Yu PS Chen C-F A new method to measure the semantic similarity of GO terms Bioinformatics 2007 23 10 1274 1281 10.1093/bioinformatics/btm087 17344234

[B20] Tao Y Sam L Li J Friedman C Lussier YA Information theory applied to the sparse gene ontology annotation network to predict novel gene function Bioinformatics 2007 23 13 i529 538 10.1093/bioinformatics/btm195 17646340 PMC2882681

[B21] Lin D An information-theoretic definition of similarity15th International Conference on Machine Learning (ICML'98): 1998; Madison, Wisconson 1998 Morgan Kaufmann 296 304

[B22] Yu H Jansen R Stolovitzky G Gerstein M Total ancestry measure: quantifying the similarity in tree-like classification, with genomic applications Bioinformatics 2007 23 16 2163 2173 10.1093/bioinformatics/btm291 17540677

[B23] Resnik P Using information content to evaluate semantic similarity in a taxonomy14th International Joint Conference on Artificial Intelligence (IJCAI-95): August 20-25, 1995 1995; Montreal, Canada 1995 448 453

[B24] Resnik P Semantic similarity in a taxonomy: An information-based measure and its application to problems of ambiguity in natural language Journal of Artificial Intelligence Research 1999 11 95 130

[B25] del Pozo A Pazos F Valencia A Defining functional distances over Gene Ontology BMC Bioinformatics 2008 9 1 50 10.1186/1471-2105-9-50 18221506 PMC2375122

[B26] Mulder NJ Apweiler R Attwood TK Bairoch A Bateman A Binns D Bradley P Bork P Bucher P Cerutti L InterPro, progress and status in 2005 Nucleic Acids Research 2005 33 Database D201 205 15608177 10.1093/nar/gki106 PMC540060

[B27] Hong EL Balakrishnan R Dong Q Christie KR Park J Binkley G Costanzo MC Dwight SS Engel SR Fisk DG Gene Ontology annotations at SGD: new data sources and annotation methods Nucleic Acids Research 2007 36 Database D577 D581 10.1093/nar/gkm909 17982175 PMC2238894

[B28] Kanapin A Batalov S Davis MJ Gough J Grimmond SM Kawaji H Magrane M Matsuda H Schonbach C Teasdale RD Mouse Proteome Analysis Genome Research 2003 13 6B 1335 1344 10.1101/gr.978703 12819131 PMC403658

[B29] Zhou X Zu Z EasyGO: Gene Ontology-based annotation and functional enrichment analysis tool for agronomical species BMC Genomics 2007 8 246 10.1186/1471-2164-8-246 17645808 PMC1940007

[B30] Zheng Q Wang X-J GOEAST: a web-based software toolkit for Gene Ontology enrichment analysis Nucleic Acids Res 2008 36 Web Server 358 363 10.1093/nar/gkn276 PMC2447756 18487275

[B31] Chu S DeRisi J Eisen M Mulholland J Botstein D Brown PO Herskowitz I The transcriptional program of sporulation in budding yeast Science 1998 282 5389 699 705 10.1126/science.282.5389.699 9784122

[B32] Bland JM Altman DG Multiple significance tests: the Bonferroni method British Medical Journal 1995 310 170 7833759 10.1136/bmj.310.6973.170 PMC2548561

[B33] Benjamini Y Hochberg Y Controlling the False Discovery Rate: a Practical and Powerful Approach to Multiple Testing Journal of the Royal Statistical Society B 1995 57 1 289 300

