# Supplementary material for: Automatic, context-specific generation of Gene Ontology slims
Source: BMC Bioinformatics. 2010 Oct 7;11:498. doi: 10.1186/1471-2105-11-498 (PMC3098080; doi:10.1186/1471-2105-11-498)
Supplement: Additional file 5 — Supplementary Methods. This file contains implementation details for the information contents calculation algorithm. [file 1471-2105-11-498-S5.DOC]

# Supplementary Methods

The implementation details of graph slim algorithm (main manuscript, Section 2.4) are described in Figure S1:

| Pre-condition:  GO Graph *G,* Annotation subset *A,* Root *r*  *r*∈*G*; Leaf nodes *L*  *L*⊂*G* Number of annotations *δ* per node *n*  *n*∈G, Threshold τ, bitmap table *B* and dynamic programming table *D*. |
| --- |
| STEP 1: For each leaf node *L* in *G*  STEP 1.a: Search for all possible paths using bottom up approach from leaf nodes to *r*.  STEP 1.b: Update list of each node's child list with their corresponding annotation count *A_n_* in a dynamic programming table.  END  STEP 2: For each node *n* in *G*  STEP 2.a: Compute *β_n_* using (3) (Section 2.4) for each node in *G* using bottom up traversal and store in a dynamic programming table *D*.  STEP 2.b: Compute information gain *P_n_* for each node *n* using (2).  STEP 2.c: Calculate information loss *Θ_n_* for each node *n* using (4).  STEP 2.d: Compute information content *I_n_* of each node using (1).  END  STEP 3: Select a set of nodes *χ* with *I* > τ |
| **Post-condition:** A set of GO terms *χ* with *I* > τ |

Figure S.1 Algorithm used to select a set of GO terms where information content I > τ

Equation numbers referred to here are drawn from the Methods Section 2.4 of the main paper.

The algorithm consists of three major phases. In the first phase, it constructs a dynamic programming table *D* by traversing all the possible paths from leaf nodes to the root node. It does so by iteratively selecting each of the leaf nodes and then traverses all the possible paths up to the root node (Figure S.1, Step 1). An entry in *D* contains the number of annotations *A_n_* for a node *n*, the number of annotations of children nodes, and an entry of a bitmap *B,* which is a list of child nodes contributions to the annotation count. For each visit of a node from leaf nodes, the entries in the dynamic programming table *D* are updated. During this process, bitmap table entry is the frequently updated. The entry contains a snapshot of direct and indirect children (grand children) of a node (Figure S.1, Step 1.b).

Once all the possible paths to the root node from leaf nodes are traversed and the construction of a dynamic programming table is up to date, the second phase begins. In the second phase information contents of each node are calculated (Figure S.1, Step 2) using equation (1). To calculate the information contents of a node *n,* first of all *β_n_* is calculated (Figure S.1, Step 2.a) using equation (3). Once the value of *β_n_* is computed, it is used to calculate the information gain *P_n_* (Step 2.b) using equation (2). After that, the algorithm calculates information loss *Θ_n_* (Figure S.1, Step 2.c) using (4). The final step (Step 2.d) of this phase is then to compute the information content *I_n_* of a node *n* using (1).

In the third phase, after all the information contents values are calculated, terms exceeding the threshold τ are selected (Figure S.1, Step 3).

An example of a small Directed Acyclic Graph (DAG) graph is presented in Figure S.2. To compute the information contents of each node, our recursive algorithm starts from leaf nodes 1, 2 and 3 iteratively and traverses all the possible paths to the root node (Figure S.1, Step 1). Initially the algorithm starts updating dynamic programming table entries of all the nodes in the path from node 1 to the root node using bottom up traversal. During this step it also updates the bitmap table entries of each node. Bitmap table *B* guarantees that the annotations of a child node are only considered once in calculating *β_n_*, to counter the fact that a node could be traversed more than once. For example, Node 4 is visited twice on paths from leaf node 1 to the root node (Figure S.2) once on the path 1🡪 **4** 🡪 9 🡪 11 and second time on the path 1🡪 **4** 🡪 10 🡪 11. The value *β_4_* is updated and stored in the dynamic programming table on each visit and considered complete only when all the paths are traversed in the graph, however the annotations of term 1 contribute only once to *β_4_*. After computing *β_4_*, the penalty term Θ*_4_* (information lost if node 4 is removed from the graph) is computed by Step 2.c and then information content is calculated in Step 2.d.

Figure S.2 An example of a DAG

Figure S.3 Graph traversal snapshot, starting from leaf nodes 1, 2 and 3 to common ancestral node 11.

To guide the reader Table S.4 shows step wise information content calculations of the example ontology graph presented in Figure S.4. For the sake of simplicity$\lambda=1$is used in the following calculations. The calculations once again show an interesting pattern. For example, node 4 and 5 in Figure S.4 have low *I_n_* values and will be removed if the threshold is set to 0.08. On the other hand Node 1, which has annotation count *A­_n_*= 0 still has the highest *I_n_* value because of high information gain *P_n_*.

**Figure S.4:** An example of an ontology graph

**Table S.1:** Information content calculations for the graph presented in S.4

| **Node** | ***A_n_*** | **Child Nodes** | $\sum_{i=1}^{\zeta} \beta_{i}\text{ }$ **in Equation (3)** | ***β_n_*** | ***P_n_*** | $\alpha$ | $\Theta_{n}=\lambda\sum_{i=1}^{\alpha} P_{i}\log_{k}(\alpha)$**Equation (4)** | $\text{I}_{\text{n}}$ |
| --- | --- | --- | --- | --- | --- | --- | --- | --- |
| 1 | 0 | {2,3,4,5} | 1159 | 1159 | 1 | 4 | 0.7109 | 0.2890 |
| 2 | 100 | {4,5} | 102 | 202 | 0.0880 | 2 | 0.0017 | 0.0862 |
| 3 | 957 | {4,5} | 102 | 1059 | 0.0880 | 2 | 0.00172 | 0.0862 |
| 4 | 100 | {5} | 2 | 102 | 0.0017 | 1 | 0 | 0.0017 |
| 5 | 2 | Null | 0 | 2 | 0 | 0 | 0 | 0 |
